# Increase of aqueous inflammatory factors in macular edema with branch retinal vein occlusion: a case control study

**DOI:** 10.1186/1476-9255-7-44

**Published:** 2010-08-26

**Authors:** Hidetaka Noma, Hideharu Funatsu, Tatsuya Mimura, Katsunori Shimada

**Affiliations:** 1 Department of Ophthalmology, Yachiyo Medical Center, Tokyo Women's Medical University, Chiba, Japan; 2 Department of Ophthalmology, University of Tokyo Graduate School of Medicine, Tokyo, Japan; 3 Department of Hygiene and Public Health II, Tokyo Women's Medical University, Tokyo, Japan

## Abstract

**Background:**

This study investigated whether soluble intercellular adhesion molecule-1 (sICAM-1) has a role in the pathogenesis of macular edema associated with branch retinal vein occlusion (BRVO) together with vascular endothelial growth factor (VEGF).

**Methods:**

A retrospective case control study was performed in 22 patients with BRVO and macular edema, as well as 10 patients with nonischemic ocular diseases as the control group. Retinal ischemia was evaluated by measuring the area of capillary non-perfusion with Scion Image software, while the severity of macular edema was examined by optical coherence tomography. Aqueous humor samples were obtained during the performance of combined vitrectomy and cataract surgery. sICAM-1 and VEGF levels in aqueous humor and plasma specimens were determined by enzyme-linked immunosorbent assay.

**Results:**

Aqueous humor levels of sICAM-1 (median: 6.90 ng/ml) and VEGF (median: 169 pg/ml) were significantly elevated in BRVO patients compared with the control group (median: 3.30 pg/ml and 15.6 pg/ml, respectively) (*P *= 0.005 and *P *< 0.001, respectively). The aqueous humor level of sICAM-1 was significantly correlated with that of VEGF (*P *= 0.025). In addition, aqueous levels of both sICAM-1 and VEGF were correlated with the size of the non-perfused area of the retina in BRVO patients (*P *= 0.021 and *P *< 0.001, respectively). Furthermore, aqueous levels of sICAM-1 and VEGF were both correlated with the severity of macular edema (*P *= 0.020 and *P *= 0.005, respectively).

**Conclusions:**

Both sICAM-1 and VEGF may be involved in the pathogenesis of macular edema associated with BRVO. Measurement of aqueous humor sICAM-1 levels may be useful for assessment of BRVO patients with macular edema, in addition to measurement of VEGF.

## Background

Branch retinal vein occlusion (BRVO) is a common retinal vascular disease that often results in macular edema, which is the most frequent cause of visual impairment in patients with BRVO [1,2]. We previously reported that vascular endothelial growth factor (VEGF) plays an important role in macular edema associated with BRVO and in breakdown of the blood-retinal-barrier (BRB). In BRVO patients with macular edema, levels of various molecules in the aqueous humor have been measured in the context of anti-VEGF therapy [3]. Among these molecules, VEGF has been reported to be the major contributor to macular edema associated with BRVO [4]. However, the pathogenesis of BRVO is complex, so measurement of VEGF alone may not provide enough information about the disease process or the response to treatment, including vitrectomy, anti-VEGF therapy, and intravitreous injection of triamcinolone acetonide.

Leukocytes also have a role in increasing vascular permeability along with VEGF. Leukocytes accumulate in the tissues through interactions with vascular endothelial cells that are mediated by intercellular adhesion molecule-1 (ICAM-1) expressed on the vascular endothelium [5]. VEGF has been reported to increase ICAM-1 expression by cultured endothelial cells [6]. Although a significant increase of various inflammatory molecules (interleukin-6, interleukin-8, inducible protein-10, and monocyte chemotactic protein-1) was not observed in the aqueous humor of BRVO patients compared with control patients [3], it is possible that these inflammatory molecules could promote adhesion of leukocytes to the vascular endothelium by regulation of ICAM-1 [7-9]. Thus, the relation between aqueous levels of ICAM-1 or VEGF and macular edema associated with BRVO remain unclear. Accordingly, we measured the aqueous levels of soluble ICAM-1 (sICAM-1) and VEGF in BRVO patients with macular edema to investigate the influence of these molecules on macular edema in the setting of BRVO.

## Methods

### Subjects

Undiluted aqueous humor samples were harvested at the start of combined vitrectomy and cataract surgery after informed consent was obtained from each subject following an explanation of the purpose and potential adverse effects of the procedure. This study was performed in accordance with the Helsinki Declaration of 1975 (1983 revision) and the institutional review board also approved the protocol for collection of aqueous humor and blood samples. Consecutive patients with BRVO presenting to the Department of Ophthalmology at Tokyo Women's Medical University between November 2006 and June 2009, were screened according to the following inclusion and exclusion criteria. The inclusion criteria were: (1) clinically detectable diffuse macular edema or cystoid macular edema (CME), (2) best-corrected visual acuity worse than 20/40 in patients scheduled for combined vitrectomy and cataract surgery, and (3) prolonged macular edema even after photocoagulation. Significant macular edema was defined as retinal thickening of one optic disc area or greater in size that involved the fovea [10]. Exclusion criteria were: (1) previous ocular surgery or intravitreous injection of anti-VEGF agents and triamcinolone acetonide, (2) diabetes mellitus and diabetic retinopathy, (3) iris rubeosis, and (4) a history of ocular inflammation or vitreoretinal disease. Twenty-two BRVO patients and 10 patients with nonischemic ocular diseases (control group) were enrolled (Table 1). The 10 control patients included 3 with a macular hole and 7 with an epiretinal membrane; none of them had associated proliferative vitreoretinopathy. The BRVO patients comprised 11 men and 11 women aged 69.3 ± 8.1 years (mean ± SD), while the control group included 2 men and 8 women aged 68.1 ± 6.9 years (Table 1). The mean duration of BRVO was 5.0 ± 2.3 months (range: 3 - 12 months). Preoperative photocoagulation was performed in 4 eyes (mean: 243 shots; range: 134 to 403), being done within 3.5 ± 1.0 months (range: 3 to 5 months) before surgery.

**Table 1:** Profile of the BRVO and control groups

	BRVO		Control	*P *value
*P *value	**No.**^**† **^**(N = 22)**		**No.**^**† **^**(N = 10)**	
Gender				
Female	11 (50.0%)		8 (80.0%)	0.109
Male	11 (50.0%)		2 (20.0%)	
Age (yr)	69.3 ± 8.1^‡^		68.1 ± 6.9^‡^	0.688
Hypertension				
No	8 (36.4%)		9 (90.0%)	0.005
Yes	14 (63.6%)		1 (10.0%)	
Duration of BRVO (months)	5.0 ± 2.3^‡^			

^†^Number of patients with data.

^‡^Mean ± standard deviation (SD).

NS = not significant.

BRVO = branch retinal vein occlusion.

### Fundus findings

Patients were evaluated by careful biomicroscopic examination using a fundus contact lens. Fundus findings were confirmed preoperatively by standardized fundus color photography. Fluorescein angiography was performed with a Topcon TRC-50EX fundus camera, an image-net system (Tokyo Optical Co Ltd., Japan), and a preset lens with a slit-lamp.

Both preoperative and operative fundus findings were recorded for each subject. A masked grader independently assessed ischemic retinal vascular occlusion by examining the fluorescein angiograms. The ischemic region of the retina was measured with the public domain Scion Image program, as reported previously [11-13]. On digital fundus photographs, the disc area was outlined with a cursor and then measured, and the same was done for the non-perfused area. Any sites of retinal photocoagulation were excluded when calculating the size of the non-perfused area. The severity of retinal ischemia was assessed as the non-perfused area divided by the disc area.

Optical coherence tomography (OCT) was performed in each subject within 1 week before vitrectomy, employing an instrument from Zeiss-Humphrey Ophthalmic Systems (Dublin, California, USA). The fundus was scanned with the measuring beam focused on horizontal and vertical planes crossing the center of the fovea, which was located by examination of the fundus photograph and by each patient's fixation. All of the subjects were able to fix on the central landmark during the examination. Cross-sectional images were collected by a single experienced examiner, who continued each examination until highly reproducible scans were obtained. The thickness of the central fovea was defined as the distance between the inner limiting membrane and the retinal pigment epithelium (including any serous retinal detachment), and was automatically measured by computer software. The thickness of the neurosensory retina was defined as the distance between the inner and outer neurosensory retinal surfaces [14], and the severity of macular edema was graded from the measured retinal thickness. In the BRVO patients, the average preoperative retinal thickness was 596 ± 182 μm, with a range of 302 to 975 μm.

### Sample collection

Combined vitrectomy and cataract surgery were performed at the Department of Ophthalmology at Tokyo Women's Medical University (Chiba, Japan).

Samples of undiluted aqueous humor (100-200 μl) were collected into sterile tubes at the start of surgery and were rapidly frozen at -80°.

Blood samples were simultaneously collected and centrifuged at 3,000 g for 5 minutes to obtain plasma, which was aliquoted and stored at -80° until assay.

### Measurement of sICAM-1 and VEGF

Levels of sICAM-1 and VEGF were measured in aqueous humor and plasma with enzyme-linked immunosorbent assay kits for human VEGF and sICAM-1 (R&D Systems, Minneapolis, MN, USA) [15]. The VEGF kit detected two of the four VEGF isoforms (VEGF_121 _and VEGF_165_). The levels of these factors in the aqueous humor and plasma were within the detection ranges of both assays, with the minimum detectable concentration being 15.6 pg/ml for VEGF (intra-assay coefficient of variation (CV): 5.5%; inter-assay CV: 6.9%) and 3.3 ng/ml for sICAM-1 (intra-assay CV: 5.4%; inter-assay CV: 7.7%).

### Statistical analysis

All analyses were performed with SAS System 9.1 software (SAS Institute Inc., Cary, North Carolina, USA). Data are presented as the mean ± SD or as the median with interquartile range or frequency. Student's *t*-test was employed to compare normally distributed unpaired continuous variables between the two groups and the Mann-Whitney U test was used for other variables with a normal distribution. The paired *t*-test or Wilcoxon's signed-rank test was employed to compare paired continuous variables. To examine the relations between sICAM-1 or VEGF and the severity of BRVO, Spearman's rank-order correlation coefficients were calculated and multiple linear regression analysis was also employed. Two-tailed P values of less than 0.05 were considered to indicate statistical significance.

## Results

### Aqueous humor levels of sICAM-1 and VEGF

**Figure 1:**
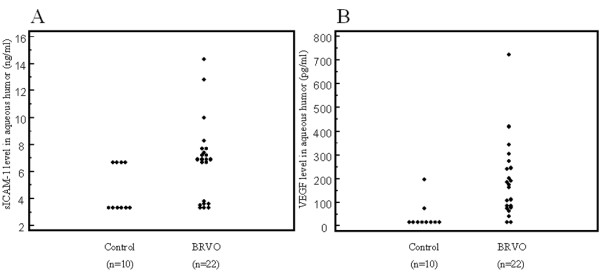
(A) Soluble intercellular adhesion molecule-1 (sICAM-1) concentrations in the aqueous humor of non-ischemic control patients and branch retinal vein occlusion (BRVO) patients with macular edema (*P = 0.005); (B) Vascular endothelial growth factor (VEGF) concentrations in the aqueous humor of non-ischemic control patients and BRVO patients with macular edema (*P < 0.001).

**Figure 2:**
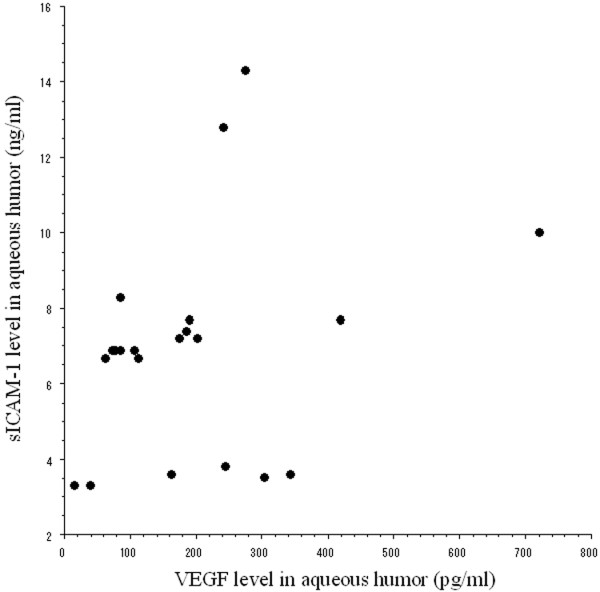
**Relation between the aqueous humor levels of soluble intercellular adhesion molecule-1 (sICAM-1) and vascular endothelial growth factor (VEGF) and in patients with macular edema and branch retinal vein occlusion (BRVO) (ρ = 0.49, *P *= 0.025)**.

Aqueous levels of sICAM-1 (median [range]) were significantly elevated in the BRVO patients (6.90 pg/ml [3.60-7.70]) compared with the control subjects (3.30 pg/ml [3.30-6.70]; *P *= 0.005; Figure 1A). Aqueous levels of VEGF were also significantly higher in the patients with BRVO (169 pg/ml [78.2-244]) than in the controls (15.6 pg/ml [15.6-15.6]; *P *< 0.001; Figure 1B). In addition, aqueous levels of sICAM-1 and VEGF were significantly correlated in the BRVO patients (Ρ = 0.49, *P *= 0.025; Figure 2).

### Influence of sICAM-1 and VEGF on ischemia and macular edema in BRVO

**Figure 3:**
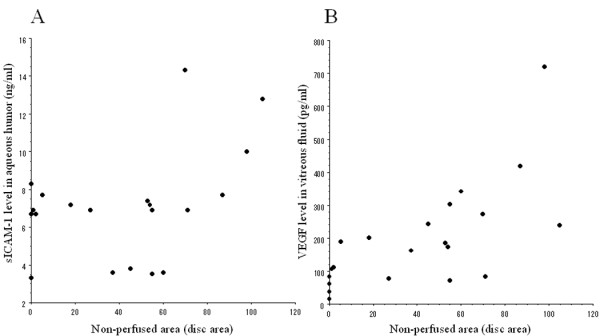
**Correlation between the severity of retinal ischemia and the aqueous humor levels of sICAM-1 and VEGF**. Retinal ischemia was evaluated by measuring the non-perfused area using Scion Image. The aqueous humor levels of sICAM-1 and VEGF were significantly correlated with the nonperfused area of the retina (sICAM-1, ρ = 0.51, *P *= 0.021; VEGF, ρ = 0.77, *P *< 0.001)

**Figure 4:**
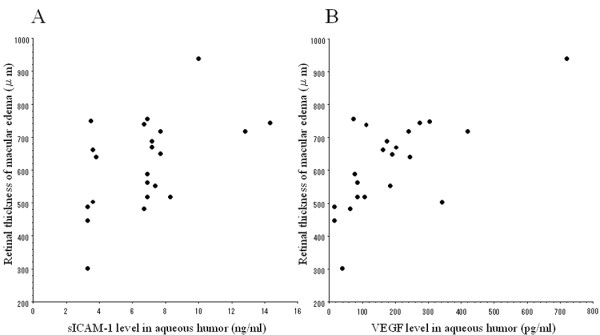
**Correlation between the severity of macular edema and the aqueous levels of sICAM-1 and VEGF**. The severity of macular edema was evaluated by optical coherence tomography. Aqueous humor levels of sICAM-1 and VEGF were significantly correlated with the retinal thickness at the central fovea (sICAM-1, ρ = 0.51, *P *= 0.020; VEGF, ρ = 0.61, *P *= 0.005).

In the BRVO patients, aqueous humor levels of both sICAM-1 and VEGF were significantly correlated with the nonperfused area of the retina (sICAM-1, ρ = 0.51, *P *= 0.021; VEGF, ρ = 0.77, *P *< 0.001; Figure 3A and 3B). In addition, aqueous levels of both sICAM-1 and VEGF were significantly correlated with the retinal thickness at the central fovea (sICAM-1, ρ = 0.51, *P *= 0.020; VEGF, ρ = 0.61, *P *= 0.005; Figure 4A and 4B). There was no significant difference of sICAM-1 or VEGF levels between the 4 patients (4 eyes) who received preoperative retinal photocoagulation and the 18 patients (18 eyes) without retinal photocoagulation (data not shown, *P *= 0.349 and *P *= 0.217, respectively). The aqueous level of sICAM-1 was not significantly correlated with either the extent or the timing of retinal photocoagulation in the 4 treated patients (ρ = 0.20, *P *= 0.729 and ρ = 0.65, *P *= 0.348, respectively). The aqueous VEGF level also showed no significant correlation with the extent or timing of photocoagulation (ρ = 0.40, *P *= 0.488 and ρ = 0.63, *P *= 0.365, respectively).

### Aqueous and plasma levels of sICAM-1 and VEGF

In BRVO patients, the aqueous level of VEGF was significantly higher than its plasma level (15.6 pg/ml [15.6-42.1]) (*P *< 0.001), while the aqueous level of sICAM-1 was significantly lower than its plasma level (381 pg/ml [316-423]) (*P *< 0.001). No correlations were observed between the aqueous and plasma concentrations of these molecules (sICAM-1, ρ = -0.04, *P *= 0.837; VEGF, ρ = 0.10, *P *= 0.993). There were also no significant correlations between the aqueous level of sICAM-1 and the age of the subjects, presence of hypertension, or duration of BRVO (data not shown, p = 0.166, p = 0.865, and p = 0.216, respectively). Furthermore, there were no significant correlations between the aqueous level of VEGF and these variables (data not shown, p = 0.548, p = 0.785, and p = 0.606, respectively).

## Discussion

The major findings of this study were as follows: (1) aqueous humor levels of VEGF and sICAM-1 were significantly higher in BRVO patients with macular edema than in control patients, (2) the aqueous levels of both VEGF and sICAM-1 were significantly correlated with the nonperfused area of the retina in BRVO patients, (3) aqueous levels of both VEGF and sICAM-1 were significantly correlated with the retinal thickness at the central fovea in BRVO patients, and (4) aqueous levels of VEGF and sICAM-1 showed a significant positive correlation with each other. These findings suggest that both VEGF and sICAM-1 may play an important role in macular edema associated with BRVO and breakdown of the BRB.

In this study, we demonstrated that the aqueous level of sICAM-1 was significantly correlated with the nonperfused area of the retina. This finding is supported by previous reports that the expression of ICAM-1 mRNA and protein is increased by retinal ischemia [16,17]. Therefore, changes of the intraocular ICAM-1 may influence the development of macular edema associated with BRVO. Increased ICAM-1 expression, leads to an increase of the rolling and adhesion of leukocytes to vein wall. After retinal vein occlusion, leukocytes are reported to show an increase of rolling and adhesion that leads to stagnation of flow [18]. Leukocytes also release substances that can damage endothelial cells and thus increase leakage from the retinal microvessels. In this context, it was reported that moderately elevated ICAM-1 expression reduces endothelial cell barrier function and that higher ICAM-1 expression affects cell junctions [19]. Macular ischemia associated with BRVO and chronic trapping of leukocytes in the retinal capillaries may promote the increased production of factors like VEGF that increase vascular permeability [6], thus leading to the development/progression of macular edema.

We also found that the aqueous level of sICAM-1 was significantly correlated with the retinal thickness at the central fovea, and that aqueous levels of sICAM-1 were significantly correlated with those of VEGF. It has been reported that VEGF increases the production of ICAM-1 by capillary endothelial cells in a dose-dependent and time-dependent manner, and that intravitreal injection of VEGF at pathophysiologically relevant concentrations increases ICAM-1 protein and mRNA levels in the retinal vasculature [6]. It has been suggested that VEGF-induced breakdown of the BRB is partly dependent on leukocytes, because inhibition of ICAM-1 expression prevents the breakdown of this barrier in the eyes of rats treated with VEGF [20]. Blocking of sICAM-1 with a neutralizing antibody was also reported to suppress both retinal leukostasis and breakdown of the BRB [20]. Thus, VEGF increases ICAM-1 expression by capillary endothelial cells *in vitro *and by the retinal vasculature *in vivo*, so that an increase of VEGF production may be responsible for increasing retinal ICAM-1 expression that mediates adhesion of leukocytes to the retinal vessels of BRVO patients. On the other hand, although various inflammatory molecules (IL-6, IL-8, IP-10, and MCP-1) have been suggested to promote leukocyte adhesion to the vascular endothelium via regulation of ICAM-1 [7-9], significantly increased concentrations of these inflammatory molecules have not been found in aqueous humor samples of BRVO patients compared with control patients [3], and the aqueous level of IL-6 is not significantly correlated with the retinal thickness at the central fovea [11]. Taken together, the results of this study and such findings suggest that measurement of sICAM-1 (and VEGF) in the aqueous humor may be more useful than these other inflammatory molecules for monitoring BRVO patients with macular edema. However, our sample size was small, so a larger prospective investigation will be required to confirm the influence of these molecules on macular edema associated with BRVO.

Recently, in the Standard Care vs Corticosteroid for Retinal Vein Occlusion (SCORE) study [21], intravitreal injection of triamcinolone acetonide was reported to improve visual acuity and macular edema after 12 months in patients with BRVO, although there was no difference of visual acuity between the standard care group (grid photocoagulation) and the triamcinolone group. Injection of triamcinolone acetonide may reduce production of ICAM-1 [22-24], thus decreasing leukocyte adhesion and avoiding breakdown of the BRB and increased vascular permeability [5,20]. Our results taken together with such reports suggest that sICAM-1 could be a potential target for preventing an increase of vascular permeability in BRVO patients with macular edema. Thus, measuring aqueous sICAM-1 levels may help to select the treatment strategy for BRVO-associated macular edema. If the aqueous level of sICAM-1 is high, patients should be considered for both anti-VEGF therapy and intravitreal injection of triamcinolone acetonide.

The present study demonstrated that there was no significant difference of aqueous sICAM-1 and VEGF levels between 4 eyes that received preoperative laser photocoagulation and 18 eyes without retinal photocoagulation. In addition, aqueous levels of sICAM-1 and VEGF showed no significant correlation with the extent or timing of retinal photocoagulation in the 4 eyes that received preoperative treatment. Musashi et al. [25] reported that the number of leukocytes showing behavior such as rolling and infiltration was increased from 12 hours after laser photocoagulation, but returned to baseline by 48 hours, and that retinal ICAM-1 mRNA expression was also upregulated in pigmented male rats after laser photocoagulation. These findings suggest that upregulation of ICAM-1 mRNA may occur transiently from about 12 to 48 hours after photocoagulation. Laser photocoagulation has also been reported to increase the expression of VEGF and transcription factors by cultured human retinal pigment epithelial (RPE) cells, with upregulation of VEGF from 6 hours to 72 hours [26]. In contrast, Itaya et al. [27] reported that the maximum VEGF level was detected on day 3 *in vivo*, coinciding with the peak of macrophage infiltration. Furthermore, changes of retinal VEGF mRNA expression in miniature pigs after laser photocoagulation were confined to RPE cells, with reduced expression immediately after photocoagulation and normalization by 42 days [28]. Together with our results, these reports suggest that retinal ICAM-1 and/or VEGF expression only shows a transient increase after photocoagulation, and that ICAM-1/VEGF levels return to baseline relatively rapidly. Because the average interval from laser photocoagulation to vitrectomy was 3.5 months in our study, laser therapy is unlikely to have influenced the levels of sICAM-1 and/or VEGF. However, further investigation will be needed to clarify the relations between the extent and timing of photocoagulation and aqueous levels of sICAM-1 and/or VEGF.

## Conclusions

In conclusion, we found that the aqueous humor level of sICAM-1 was significantly correlated with the retinal thickness at the central fovea, and that it was also significantly correlated with the aqueous humor level of VEGF. These results suggest that measurement of aqueous sICAM-1 levels may also be useful for monitoring BRVO patients with macular edema, in addition to measurement of VEGF.

## Financial support

None.

## Competing interests

The authors declare that they have no competing interests.

## Authors' contributions

All authors have read and approved the final manuscript. HN, and HF were involved in the design and conduct of the study. Collection and management of the data were done by HN, while analysis and interpretation of the data were performed by HN, TM, and KS. Preparation of the first draft of the manuscript was done by HN, and review and approval of the manuscript was performed by HF, and TM.
